# Dynamic evolution of rocky desertification and vegetation restoration and analysis of driving forces in Southwest Karst Region from 2000 to 2020

**DOI:** 10.1371/journal.pone.0332644

**Published:** 2025-11-14

**Authors:** Qing Yang, Jinping Chen, Guangbin Yang, Hang Xie, Man Li, Junying Sun

**Affiliations:** 1 School of Geography and Environmental Science, Guizhou Normal University, Guiyang, China; 2 School of Resources and Environmental Engineering, Anshun University, Anshun, China; 3 Key Laboratory of Remote Sensing Applications in Mountain Resources and Environment, Guiyang, Guizhou, China; 4 School of Soil and Water Conservation, Southwest Forestry University, Kunming, China; 5 The First Surveying and Mapping Institute Of Guizhou Province, Guiyang, China; 6 The Second Surveying and Mapping Institute Of Guizhou Province, Guiyang, China; HUN-REN Centre for Ecological Research, HUNGARY

## Abstract

The karst region in southwestern China is particularly prominent and has become a core issue constraining ecological environment restoration and sustainable development in this area. This study utilized long-term remote sensing data to reveal the spatial pattern evolution characteristics of rocky desertification in the region in 2000, 2010, and 2020. Meanwhile, it analyzed the dynamic trend of vegetation coverage recovery in the area from 2000 to 2020, as well as the analysis of related factors. The results showed that the spatial distribution of the Normalized Difference Vegetation Index (NDVI) remained highly clustered, though the clustering gradually weakened over time. When NDVI exceeded 0.6, the probability of rocky desertification reversal increased. Currently, a core contradiction of “quantity increases but quality stagnates” exists in regional vegetation cover, characterized by a continuous rise in NDVI mean values coexisting with reduced spatial clustering. This phenomenon reflects the evolution of vegetation patterns under the combined effects of ecological engineering interventions, adjustments in human-land relationships, and constraints of karst landforms. Through factor analysis, slope and humidity were identified as key factors influencing vegetation restoration. The findings provide an important theoretical foundation and practical reference for targeted rocky desertification management, optimization of ecological restoration projects, and coordinated human-land development in karst regions.

## 1. Introduction

In Karst areas, the weathering and soil formation rates of carbonate rocks are low, leading to poor soil, limited vegetation growth, and a fragile ecosystem [1,2]. The physical properties and soil-forming conditions of carbonate rocks, coupled with human activities like inappropriate land reclamation, make these areas highly prone to rocky desertification [3,4]. Karst rocky desertification refers to a land degradation process occurring in the fragile subtropical karst environments [5,6]. The karst region of southwestern China is one of the world’s most typical areas of karst geomorphological development and one of the regions with the most severe ecological challenges. This phenomenon is manifested as vegetation degradation and soil/water loss under the combined effects of natural factors and human activities [7], This is accompanied by exacerbated soil erosion and a marked decline in vegetation cover [8]. This distinctive ecological degradation pattern leads to heightened system vulnerability, creating a multi-dimensional environmental crisis that spans geological, ecological, and social dimensions.

To address this ecological challenge, a series of large-scale ecological restoration projects have been implemented since the early 21st century. Currently, geographic information technology has matured, and remote sensing technology provides abundant data sources for research. From global and sustainable development perspectives, studies have been conducted to analyze regional land degradation and conversion phenomena along with their driving factors [9], establishing a scientific foundation for regional land restoration and sustainable management. The United Nations Sustainable Development Goals (SDGs) require quantifying vegetation proportion as a percentage of a nation’s total land area. International research has employed remote sensing technology and machine learning to monitor vegetation and analyze influencing factors [10], investigating issues such as land degradation and desertification [11]. In karst regions, comprehensive management strategies—including vegetation restoration, soil and water conservation, and industrial restructuring—advance the in-depth management of rocky desertification [12,13]. Following project implementation, regional ecological conditions have progressively improved [14]. Concurrently, significant progress has been made in understanding the interactive mechanisms linking rocky desertification with climate change, land-use transitions, and human activity intensity [15,16]. These studies have elucidated the characteristic evolutionary processes of rocky desertification driven by the synergistic effects of multiple factors. In karst regions, the Normalized Difference Vegetation Index (NDVI) is commonly employed to assess the ecological environment affected by rocky desertification. As a critical indicator of vegetation dynamics, NDVI can effectively characterize spatial and temporal vegetation changes. The analysis of long-term NDVI time series variations enables the characterization of vegetation dynamics and health conditions in specific regions, serving as a scientific basis for monitoring ecosystem rehabilitation and evaluating restoration efficacy [17]. In karst regions, vegetation growth demonstrates positive correlations with temperature and precipitation [18,19],while slope gradient and elevation tend to hinder vegetation growth and recovery rates [20,21]. The heterogeneity in rock-soil structures attributable to lithological backgrounds also serves as a critical determinant of vegetation restoration variability [22]. However, restoration practices have revealed nonlinear feedback mechanisms between carbonate rock-dominated rocky desertification processes and vegetation recovery [23–25]. When ecological thresholds are reached during remediation, risks of retrogressive succession may compromise restoration outcomes. This dynamic coupling relationship remains insufficiently elucidated in current research frameworks.

In carbonate-dominated karst fragile ecosystems, elucidating the spatiotemporal differentiation dynamics of rocky desertification evolution and deciphering the key driving mechanisms of vegetation restoration constitute pivotal scientific inquiries for addressing ecological degradation and advancing the understanding of human-environment feedback systems. Current research gaps include: (1) insufficient comparative studies on long-term vegetation recovery trajectories in typical rocky desertification areas with carbonate lithological predominance; (2) incomplete mechanistic understanding of how vegetation restoration processes respond to natural drivers and anthropogenic interventions within the context of dynamic rocky desertification evolution.

Therefore, this study integrates multi-source remote sensing data, applies GIS spatial analysis, trend analysis method and geographical detector, and combines ArcGIS for spatial overlay analysis to quantitatively analyze the spatio-temporal evolution law of rocky desertification in the karst areas of Southwest China, reveal the change law of vegetation coverage from 2000 to 2020, and clarify the driving mechanism of natural and human factors on rocky desertification and vegetation restoration. In order to reveal the response mechanism of various influencing factors in the dynamic change process of the karst rocky desertification area dominated by carbonate rocks in the southwest, and to identify the main causes based on the recovery characteristics of the rocky desertification area, this study aims to provide spatial decision support for the zonal governance of rocky desertification and offer references for the sustainable management of the fragile karst ecosystem.

## 2. Study area and data

### 2.1 Study area

The study area encompasses eight provincial-level administrative units in southwest China: Guizhou, Yunnan, Guangxi, Sichuan, Chongqing, Hunan, Hubei, and Guangdong (21°08′–34°19′N, 97°31′–112°04′E). This region represents the world’s largest contiguous karst terrain and the most severe rocky desertification (KRD) hotspot. Characterized by a humid subtropical monsoon climate with marked transitional features, the area exhibits an annual mean temperature of 14.5–22.3°C, accumulated temperatures ≥10°C ranging from 4,500–7,500°C, and a frost-free period of 280–350 days. Annual precipitation averages 850–1,444 mm, with 60–70% concentrated during May–September, demonstrating pronounced synchronicity between rainfall and thermal regimes.

Unique geological-climatic conditions shape distinctive hydro-ecological patterns: carbonate rocks cover >71.3% of the area, fostering intense karst dissolution processes under warm-wet conditions. This results in underdeveloped surface water systems, where >90% of precipitation rapidly infiltrates into underground networks through sinkholes and fissures, creating a paradoxical hydrological phenomenon of “surface water scarcity amid abundant groundwater.” Coupled with high solar radiation (6,000 MJ/m²/yr) and potential evapotranspiration (800–1,200 mm/yr), these factors drive the formation of ecologically vulnerable landscapes typified by “shallow soils, water deficits, and rocky exposures.” Currently, KRD-affected lands span 120,300 km², accounting for 88.6% of China’s total rocky desertification area, with escalating risks of vegetation retrogressive succession.

### 2.2 Data sources and preprocessing

This study integrates multi-source datasets including vector maps, Landsat 7/8 surface reflectance data, MODIS products (land surface temperature (LST) and soil moisture), socioeconomic data (population, GDP), nighttime light (NL), meteorological data (temperature, precipitation), and topographic derivatives (slope),Data sources (Table 1).

**Table 1:** Data sources and specifications.

Data Type	Spatial Resolution	Temporal Coverage	Data Source
Vector maps	N/A	2024	https://www.tianditu.gov.cn
DEM	30m	N/A	https://www.usgs.gov/tools/national-map-viewer
Landsat7/8	30m	2000-2020	
MODIS	1000m	2000-2020	
GDP	1000m	2000-2020	www.resdc.cn
Population	1000m	2000-2020	
Precipitation	1000m	2000-2020	https://data.tpdc.ac.cn/zh-hans/data
Nighttime Light	1000m	2000-2020	https://www.ngdc.noaa.gov/

Based on China’s 1:2500000 scale topographic maps and with reference to previous research results [26], the carbonate rock areas are divided into karst areas, and the rest are non-karst areas.

To ensure consistency in the spatial resolution of the data, we sequentially performed cropping and mosaicking on the original data, and uniformly resampled all data to a 1 km resolution, ultimately forming the dataset used in this study.

## 3. Research methodology

### 3.1 Rocky desertification evaluation method

A rocky desertification evaluation index system was constructed to calculate the distribution data of rocky desertification in the southwestern karst region from 2000 to 2020. The evaluation index system is shown in Table 2, which was used to determine the spatial distribution of rocky desertification in the study area.

**Table 2:** Evaluation index system.

Classification	Rock exposure rate (%)	Vegetation Coverage (%)	Slope (°)
No Desertification	0-30	70-100	0-5
Light Desertification	30-50	60-70	5-15
Moderate Desertification	50-60	50-60	15-25
Severe Desertification	60-80	30-50	25-35
Extreme Desertification	Over 80	0-30	Over 35

The research classified rocky desertification into five severity levels: non-desertified, mild, moderate, severe, and extremely severe. High-resolution imagery combined with field surveys was employed to validate typical regions. Areas with incorrect interpretations were revised to enhance classification accuracy. Finally, ArcGIS software was utilized to organize the database, including operations such as mosaicking, merging, and removing small patches from the final output data. This process generated spatial distribution maps of rocky desertification in the southwestern karst region from 2000 to 2020.

(1) Lithology

In this study, the rock exposure rate in karst areas was obtained based on the Normalized Difference Rock Index (NDRI). The calculation formulas are as follows:


$$NVRI=\frac{\left({\text{Band}}_{\text{5}}-{\text{Band}}_{\text{3}}\right)}{\left({\text{Band}}_{\text{5}}+{\text{Band}}_{\text{3}}\right)}$$ (1)

$$f_{\text{r}}=\frac{\left(NDRI-{NDRI}_{\text{0}}\right)}{\left({NDRI}_{\text{r}}-{NDRI}_{\text{0}}\right)}$$ (2)

Specifically, ${NDRI}_{\text{0}}$ denotes the pixel value of the Normalized Difference Rock Index (NDRI) at a cumulative frequency of 1%, while ${NDRI}_{\text{r}}$ represents the pixel value of the NDRI at a cumulative frequency of 95%.

(2) Vegetation Coverage Fraction

The pixel dichotomy model based on the normalized difference vegetation index (NDVI) was used to estimate the vegetation coverage fraction (FVC) in karst areas. The calculation formula is as follows:


$$\text{NVDI}=\frac{\left(\text{NIR}-\text{R}\right)}{\left(\text{NIR}+\text{R}\right)}$$ (3)

$${\text{f}}_{\text{c}}=\frac{\left(\text{NDVI}-{\text{NDVI}}_{\text{soil}}\right)}{\left({\text{NDVI}}_{\text{veg}}-{\text{NDVI}}_{\text{soil}}\right)}$$ (4)

where ${\text{NDVI}}_{\text{soil}}$ is the NDVI value corresponding to the 5% cumulative frequency, and ${\text{NDVI}}_{\text{vge}}$ is the NDVI value corresponding to the 95% cumulative frequency [27].

(3) Slope

High-resolution DEM (Digital Elevation Model) data of the study area was collected, and slope information was extracted using ArcGIS software. In karst rocky desertification areas, sloping farmland with a slope greater than 35° is regarded as a key area for rocky desertification [2]. The software’s reclassification function was used to classify and assign grades according to the slope classification criteria set in Table 2.

### 3.2 Spatiotemporal evolution process model of rocky desertification

The single-grade rocky desertification dynamics (K) and comprehensive-grade rocky desertification dynamics (P) have become important indicators for quantitative analysis of the spatiotemporal evolution process of rocky desertification [28,29]. Their calculation methods are shown in formulas (5)–(6).


$$\text{K}=\frac{{\text{U}}_{\text{b}}-{\text{U}}_{\text{a}}}{{\text{U}}_{\text{a}}}\times \frac{\text{1}}{\text{T}}\times \text{100}\%$$ (5)

$$\text{P}=\sum_{\text{i}=\text{1}}^{\text{n}}\frac{\left(\left|{\text{U}}_{\text{bi}}-{\text{U}}_{\text{ai}}\right|\right)}{\text{S}}\times \frac{\text{1}}{\text{T}}\times \text{100}\%$$ (6)

In the formulas: K represents the dynamics of a specific rocky desertification type during the study period; Uₐ and Ubdenote the quantities of this rocky desertification type at the beginning and end of the study period, respectively; T is the number of years between the start and end of the study period; P refers to the comprehensive-grade dynamics of rocky desertification types during the study period;Uₐᵢ and Ubᵢ are the statistical areas of the i-th rocky desertification type in the study area at the start and end of the study period, respectively; S is the total land area of the study area; n represents the number of rocky desertification types.

### 3.3 Trend analysis method

Simple linear regression was used to analyze the spatial variation trend of NDVI during the vegetation growing season (April–October) in the Southwest Karst Region from 2000 to 2020, with the formula as follows:


$$\theta_{\text{slope}}=\frac{\text{n}\times \sum_{\text{i}=\text{1}}^{\text{n}}\text{i}\times {\text{x}}_{\text{i}}-\sum_{\text{i}=\text{1}}^{\text{n}}\text{i}\sum_{\text{i}=\text{1}}^{\text{n}}{\text{x}}_{\text{i}}}{\text{n}\times \sum_{\text{i}=\text{1}}^{\text{n}}{\text{i}}^{\text{2}}-{\left(\sum_{\text{i}=\text{1}}^{\text{n}}\text{i}\right)}^{\text{2}}}$$ (7)

The analysis results can reflect the characteristics of NDVI changes over time in this region. In the formula, the slope represents the change trend of the x index from 2000 to 2020, n = 1,2, … denote the years, and xi is the value of the x index for the i -th year. If the slope is positive, it indicates that NDVI shows an upward trend; if negative, it indicates a downward trend.

### 3.4 Spatial autocorrelation

Spatial autocorrelation methods were used to analyze the characteristics and distribution of spatial correlations [30]. Global and local autocorrelation methods can reflect the correlations between variables across the entire study area and the local clustering characteristics of spatial relationships [31]. Global autocorrelation and local autocorrelation are represented by the Moran I index and the Local Moran I index, respectively, with the formulas as follows:


$$\text{I}=\frac{\sum_{\text{i}=\text{1}}^{\text{n}}\sum_{\text{j}\neq \text{1}}^{\text{n}}{\text{W}}_{\text{ij}}({\text{y}}_{\text{i}}-{\text{y}}_{\text{mean}})({\text{y}}_{\text{i}}-{\text{y}}_{\text{nean}})}{{\text{S}}^{\text{2}}\cdot \sum_{\text{i}=\text{1}}^{\text{n}}\sum_{\text{j}\neq \text{1}}^{\text{n}}{\text{W}}_{\text{ij}}}$$ (8)

In the formula, yi and yj represent the attribute values of cell i and cell j, respectively; n is the number of spatial cells; Wij denotes the weight matrix established based on spatial contiguity.

The binary global autocorrelation and local autocorrelation based on the Moran’s I index can be used to describe the correlation degree of the spatial distribution of different elements, which are respectively expressed as:


$${\text{I}}_{\text{lm}}^{\text{p}}={\text{Z}}_{\text{l}}^{\text{p}}\cdot \sum_{\text{q}=\text{1}}^{\text{n}}{\text{W}}_{\text{pq}}\cdot {\text{Z}}_{\text{m}}^{\text{q}}$$ (9)

$${\text{Z}}_{\text{l}}^{\text{p}}=\frac{{\text{X}}_{\text{l}}^{\text{p}}-{\text{X}}_{\text{lmean}}}{{\text{e}}_{\text{l}}}$$ (10)

$${\text{Z}}_{\text{m}}^{\text{q}}=\frac{{\text{X}}_{\text{m}}^{\text{q}}-{\text{X}}_{\text{mmean}}}{{\text{e}}_{\text{m}}}$$ (11)

where XI is the value of attribute I of the spatial unit p; it is the value of attribute m of the spatial unit q; XImean and el are the mean value and variance of attribute I respectively; Xmmean and em are the mean value and variance of attribute m respectively.

Moran’s I value ranges from −1–1. A positive Moran’s I (I > 0) indicates positive spatial correlation, a negative Moran’s I (I < 0) indicates negative spatial correlation, and an Moran’s I of 0 indicates that attribute values are randomly distributed. This study uses GeoDa 1.14.0 software to plot Local Indicators of Spatial Association (LISA) cluster maps to reflect the spatial dependence and correlation of socioeconomic development levels. The output cluster maps include four types of spatial associations: high-high (HH) clusters, low-low (LL) clusters, high-low (HL) outliers, low-high (LH) outliers, and non-significant areas.

### 3.5 Geographical detectors

To address the issue of inconsistent resolution, the study area was divided into a 300 × 300m fishnet during the analysis of driving forces, resulting in a total of 14,650 sampling units. Using the natural break method, variables such as precipitation, surface temperature, humidity, population, GDP, nighttime light, and slope were classified into 8 categories. Sample points were extracted and input into Geographical Detectors to quantify the driving factors.

Using the Geographical Detectors model, the driving factors of spatial differentiation can be analyzed [32]. The basic principle of this model is to detect the explanatory power (q-value) of influencing factors by utilizing the relationship between within-layer local and global variances, which represents the degree to which explanatory variables affect the spatial differentiation of explained variables. Its mathematical expression is as follows:


$$\text{q}=\text{1}-\frac{\text{1}}{{n\sigma }^{\text{2}}\sum_{\text{h}=\text{1}}^{\text{L}}{\text{n}}_{\text{h}}\sigma_{\text{h}}^{\text{2}}}$$ (12)

Where q represents the explanatory power of the influencing factor: the larger the q-value, the greater the impact of this influencing factor on the degree of spatial differentiation.

## 4. Results

### 4.1 Analysis of the current status and changes in rocky desertification

Rocky desertification in China is mainly distributed in the karst regions of eight provinces and municipalities: Guizhou, Yunnan, Guangxi, Sichuan, Hunan, Guangdong, Chongqing, and Hubei. Spatially, areas with severe rocky desertification are primarily located in Yunnan and Guizhou, as well as the border region between Yunnan and Guangxi. Severe rocky desertification is concentrated in southeastern Yunnan (bordering Guangxi) and northeastern Yunnan (bordering Guizhou), with relatively less occurrence in other areas. According to the statistical data in this study, the rocky desertification area in 2010 was 136,600 km². Based on China’s second national rocky desertification survey in 2011, the area in Southwest China (eight provinces) was 120,000 km². A comparison between the monitoring results and those from proximate years in national bulletins indicates minimal discrepancies. The third national survey in 2016 recorded a total rocky desertification area of 100,700 km². Across the board, the data demonstrates a decreasing trend, which aligns with the trend observed in this study.

In 2000, the total area affected by rocky desertification in these eight regions was 154,200 km²; this decreased to 136,600 km² in 2010 and further dropped to 71,500 km² in 2020 (Table 3). By 2020, Yunnan had the largest rocky desertification area among the eight southwestern provinces and municipalities, totaling approximately 19,800 km², accounting for 27.73% of the total; followed by Guizhou with an area of about 18,900 km² (26.37%); and Guangxi Zhuang Autonomous Region with approximately 14,000 km² (19.52%). The areas of the remaining provinces were relatively small and accounted for a minor proportion.

**Table 3:** Rocky desertification area proportion in eight provinces, autonomous regions, and municipalities of Southwest China, 2000–2020.

Province	2000		2010		2020	
	Rocky Desertification Area (10,000 km²)	Proportion of Total (%)	Rocky Desertification Area (10,000 km²)	Proportion of Total (%)	Rocky Desertification Area (10,000 km²)	Proportion of Total (%)
Sichuan	0.17	12.58	0.89	6.55	0.34	4.76
Yunnan	3.62	23.48	3.57	26.18	1.98	27.73
Guizhou	3.73	15.64	3.89	28.47	1.89	26.37
Guangxi	2.41	8.61	2.50	18.31	1.40	19.52
Hunan	1.94	8.34	1.45	10.65	0.76	10.62
Hubei	1.33	1.08	0.53	3.86	0.32	4.47
Chongqing	1.29	24.15	0.70	5.16	0.41	5.68
Guangdong	0.94	6.12	0.11	0.81	0.06	0.85

### 4.2 Spatio-temporal evolution characteristics of rocky desertification, 2000–2020

#### 4.2.1 Analysis of rocky desertification changes by severity.

As shown in Table 4, provinces with larger areas of severe rocky desertification include Yunnan, Guizhou, and Sichuan, based on the composition of rocky desertification severity grades. In 2000, mild and moderate severity dominated the rocky desertification landscape: mild rocky desertification covered 60,800 km², accounting for 39.42% of the total rocky desertification area; moderate rocky desertification covered 46,400 km², accounting for 30.06% of the karst rocky desertification area.

**Table 4:** Changes and dynamic degrees of rocky desertification by severity in Southwest China’s rocky desertification areas.

Grade	Area (km²)			2000-2010		2010-2020	
	2000	2010	2020	change area	dynamic degree	change area	dynamic degree
Non-rocky Desertification	293316.00	310987.00	376045.20	17671.00	0.55%	65058.20	1.90%
Mild Rocky Desertification	60804.00	55204.00	37800.35	−5600.00	−0.84%	−17403.65	−2.87%
Moderate Rocky Desertification	46358.00	39704.00	29319.16	−6654.00	−1.30%	−10384.84	−2.38%
Severe Rock Desertification	40781.00	35115.00	4390.29	−5666.00	−1.26%	−30724.71	−7.95%
Extremely Severe Rock Desertification	6297.00	6545.00	0.00	248.00	0.36%	−6545.00	−9.09%

In 2010, mild and moderate severity remained the dominant types of rocky desertification: mild rocky desertification covered 55,200 km², accounting for 40.42% of the total rocky desertification area; moderate rocky desertification covered 39,700 km², accounting for 29.07%; extremely severe rocky desertification only covered 6,500 km² (4.79%), mainly distributed in provinces such as Guizhou, Yunnan, and Guangxi.

By 2020, the total rocky desertification area in the eight provinces and municipalities was 71,500 km², accounting for 3.66% of their combined total area. Mild and moderate severity continued to predominate: mild rocky desertification covered 37,800 km² (52.86% of the total), moderate rocky desertification covered 29,300 km² (41.00%), and severe rocky desertification only covered 4,400 km² (6.14%), still mainly distributed in Guizhou, Yunnan, Guangxi, and other provinces.

#### 4.2.2 Analysis of the evolution process of rocky desertification.

To explore the spatio-temporal evolution characteristics of rocky desertification, the land use dynamic change analysis model was introduced to study the dynamic degree of single-grade rocky desertification, comprehensive dynamic degree of rocky desertification by grade, area transfer matrix of rocky desertification, and bidirectional change rate of rocky desertification, in order to quantitatively describe its spatio-temporal evolution process,The results are shown in Table 4.

From the perspective of the dynamic degree of single-grade rocky desertification, the evolution of rocky desertification in the study area can be divided into two characteristics:

(1) Continuous positive dynamic degree, with a shrinking area, representing the evolution of non-rocky desertification;
(2) Continuous negative dynamic degree, also with a shrinking area, representing the evolution of other rocky desertification grades.

The continuous expansion of non-rocky desertification areas and the rapid decline of high-grade rocky desertification are consistent with the positive feedback mechanism of “artificial vegetation reconstruction–soil habitat improvement–reverse succession of rocky desertification grades.” Notably, the dynamic degree of all rocky desertification grades in the second stage was higher than that in the first stage, indicating that after breaking through ecological thresholds, governance projects produced an accelerated restoration effect, providing a quantitative basis for optimizing zonal governance strategies.

#### 4.2.3 Spatial differentiation patterns.

Based on the rocky desertification data from 2000 to 2020, this study constructed a transition matrix for rocky desertification, which showed that the overall severity of rocky desertification in Southwest China exhibited a downward trend. By classifying regional rocky desertification severity, areas where severity improved from poor to better were defined as restoration areas (accounting for 34.68%), areas with unchanged severity as stable areas (59.31%), and areas where severity deteriorated from better to worse as degradation areas(6.01%). Overall, the severity of rocky desertification showed a downward trend.

Spatially, restoration areas were mainly concentrated in large contiguous regions of Guizhou, Guangxi, Chongqing, and Yunnan. Degradation areas were primarily located in the border regions between Guizhou and Yunnan, as well as between Guizhou and Chongqing, with sporadic distributions in other areas.

**Fig 1:**
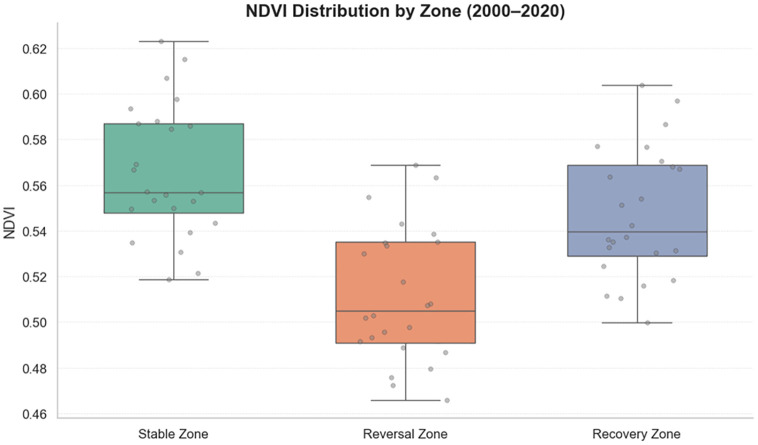
NDVI changes in stable, restoration, and degradation areas, 2000–2020.

To further compare vegetation changes across different regions, mean NDVI (Normalized Difference Vegetation Index) values were analyzed for each area. As shown in Fig 1, the results showed that stable and restoration areas had relatively higher NDVI values, indicating generally better vegetation conditions in these regions.

The degradation areas exhibited the lowest NDVI: although showing an upward trend, their degree of vegetation recovery remained weaker than the other two areas, with slower ecological restoration. Overall, the results indicated significant trends in all regions, confirming clear patterns of vegetation change in different rocky desertification areas during 2000–2020.

Overall, there is a trend of expansion in restoration areas and contraction in degradation areas for rocky desertification. The stepwise changes more clearly indicate that by identifying turning points before and after policy interventions (such as the grain-for-green program), the area affected by rocky desertification began to decline and the growth rate of NDVI significantly increased during 2010–2020. These changes align with the implementation intensity and timeline of the grain-for-green program fully launched in 2002 and the comprehensive rocky desertification control project initiated in 2007. Although the junction area of Yunnan-Guizhou-Guangxi remains the core region of severe rocky desertification, its NDVI increase is significantly higher than that of other regions, which also demonstrates the policy effectiveness of “priority restoration in key control areas” for rocky desertification regions.

### 4.3 Analysis of NDVI changes in Southwest China’s rocky desertification areas, 2000–2020

#### 4.3.1 Distribution of vegetation coverage.

Random forest regression analysis using SPSSPRO revealed a positive correlation between NDVI and rocky desertification area in 2000, 2010, and 2020. This essentially reflects the dynamic equilibrium between vegetation expansion in low-coverage areas and the reverse evolution of rocky desertification. However, this relationship exhibits nonlinear characteristics, indicating that increasing vegetation coverage alone does not necessarily reduce rocky desertification overall—there is a complex nonlinear relationship between the two.

**Fig 2:**
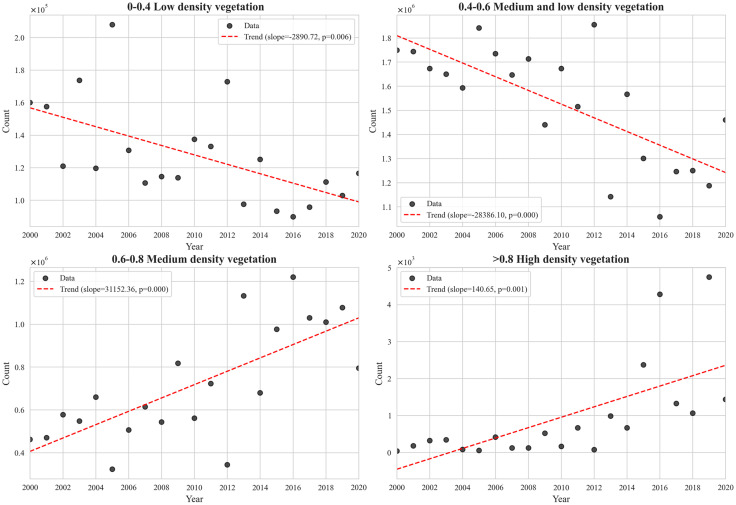
Change trends in pixel values of different vegetation coverage areas.

Therefore, to investigate the dynamic relationship between vegetation restoration processes and rocky desertification, the study classified NDVI values in the research area into different vegetation density categories based on data characteristics: low density (0–0.4), moderate-low density (0.4–0.6), moderate density (0.6–0.8), and high density (>0.8). By statistically analyzing the proportion of pixels in each vegetation coverage category from 2000 to 2020, pixel-by-pixel calculations were performed for the four vegetation coverage regions in rocky desertification areas, and the results are presented in Fig 2. Additionally, a two-tailed t-test was conducted on the regression slopes to obtain P-values for determining the statistical significance of trends: trends with P < 0.05 were considered statistically significant.

The results showed that from 2000 to 2020, areas with low and moderate-low vegetation coverage in rocky desertification regions exhibited a significant downward trend (P < 0.05), indicating a reduction in low and moderate-low density vegetation. In contrast, moderate and high vegetation coverage areas increased significantly (P < 0.05), meaning moderate and high density vegetation became more prevalent. In terms of vegetation coverage structure evolution, the proportion of low-density vegetation pixels decreased from 27.6% in 2000 to 9.3% in 2020, while high-density vegetation increased sharply from 12.8% to 34.5%. This characteristic of “low-density vegetation decline and high-density aggregation” in vegetation structure optimization showed a significant coupling with the spatio-temporal pattern of a 1.2% annual reduction in rocky desertification area.

Vegetation coverage exhibited a“double threshold effect”: when coverage exceeded the 0.6 threshold, the number of pixels in the region increased linearly. This is because vegetation canopies enhance interception efficiency of rainfall erosion and root systems strengthen soil stability, forming an ecological barrier against rocky desertification. When coverage exceeded the 0.8 threshold, the number of pixels continued to rise but at a gradually decreasing rate, suggesting that the carrying capacity of the karst habitat may be approaching its limit, leading to a diminishing marginal effect of ecological gains.

#### 4.3.2 Slope trend test.

By calculating the slope value of NDVI change over the 22-year period for each pixel and using ArcPy tools to perform pixel-level regression analysis on NDVI data from2000 to 2020, the study applied linear regression to compute the slope of NDVI changes over time (Slope) and determine trends in vegetation coverage improvement or degradation. Based on the standard least squares regression model, the output was a NDVI temporal trend layer for rocky desertification areas from 2000 to 2020.

**Fig 3:**
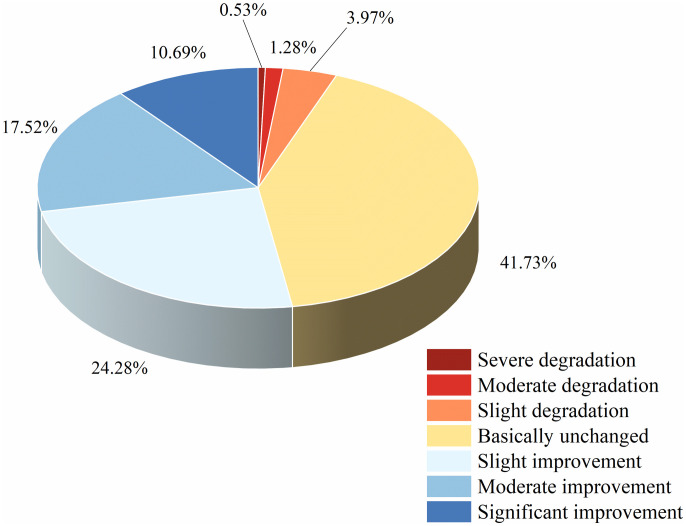
Distribution of NDVI change trends in the growing season, 2000–2020.

Following existing research [33,34] and the study’s specific context, slope values were classified into the following grades: severe degradation (slope ≤ −0.04), moderate degradation (−0.04 < slope ≤ −0.02), slight degradation (−0.02 < slope ≤ −0.01), no significant change (−0.01 < slope ≤ 0.01), slight improvement (0.01 < slope ≤ 0.02), moderate improvement (0.02 < slope ≤ 0.04), and significant improvement (slope > 0.04). The spatial distribution and area proportion of these slope values were analyzed in Fig 3.

According to the figure, areas with improved vegetation in rocky desertification regions from 2000 to 2020 were primarily concentrated in northwestern Guizhou, central Guangxi, eastern Yunnan, and northeastern Hunan. Degraded areas were mainly distributed in most of Guizhou, southeastern Chongqing, central Hunan, and most of Sichuan. Statistical analysis of the areas showing decreases and increases, as presented in Fig 3, reveals that 52.49% of the total region exhibited improved vegetation status, while degraded areas accounted for 5.78%.

### 4.4 Spatial correlation analysis

The results showed that the Global Moran’s I values (Table 5) were all greater than 0.49 (p = 0.001, z > 1.96), indicating a strong positive spatial autocorrelation and clustering of NDVI in the study area. This signifies significant spatial positive correlation and clustering of NDVI in Southwest China’s karst regions from 2000 to 2020. Although Moran’s I exhibited a gradual downward trend over the years, with a cumulative decrease of 0.034 from 2000 to 2020, its absolute value remained close to 1. This suggests that while the spatial clustering of vegetation coverage gradually weakened, the fundamental characteristic of “high aggregation” did not change.

**Table 5:** Moran’s I values, 2000–2020.

Year	Moran’s I	Z-Value	*p* Value
2000	0.534	50.777	0.001
2010	0.494	46.624	0.001
2020	0.500	46.443	0.001

This change may be associated with the gradual advancement of ecological protection policies, adjustments in the intensity of human activities, and the combined effects of climate change within the region. For example, afforestation projects implemented in some areas increased vegetation coverage, causing originally scattered vegetation patches to gradually connect—this maintained spatial aggregation to a certain extent, but the dispersed nature of these projects also led to a slight decrease in overall aggregation.

**Fig 4:**
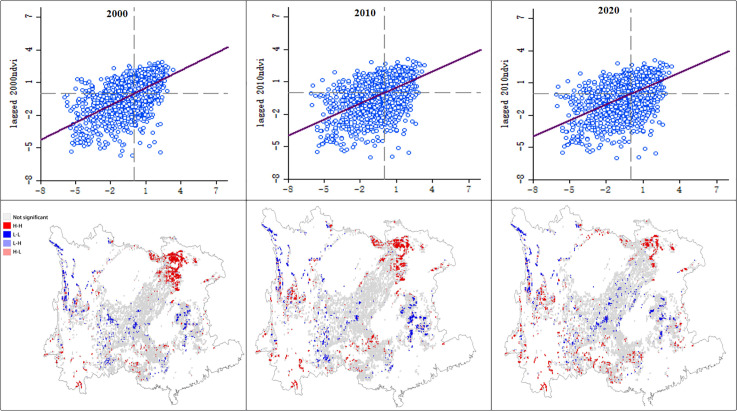
Scatter plot of NDVI Moran I and LISA cluster map (2000-2020).

As shown in Fig 4, results from local spatial autocorrelation analysis showed that Local Moran’s I values were mainly distributed in the first and third quadrants, representing high-high and low-low aggregation types with positive spatial correlation.

These clusters were primarily concentrated in regions such as Chongqing and Hubei, where unique topographies (e.g., interlaced mountains and valleys) facilitated the formation of high-high aggregation zones in suitable mountainous environments. Over time, from 2010 to 2020, high-high value clusters began to emerge in Yunnan, Guangxi, Sichuan, and other areas, while aggregation in Chongqing weakened—likely due to accelerated urban expansion, which converted some vegetated areas to development uses and disrupted original vegetation aggregation patterns. Maintaining the stability of high-value clusters to prevent forest fragmentation will be critical in the future.

Overall, low-low value areas exhibited patchy clustered distribution and showed a trend of peripheral diffusion. The decline in Moran’s I over time reflected ecological improvement in low-value areas, though positive aggregation remained dominant. Low-value areas are mostly ecologically fragile regions where vegetation was severely damaged by historical human activities such as over-cultivation and grazing. In recent years, vegetation recovery in these areas has occurred alongside the implementation of ecological compensation mechanismsand ecological restoration projects. In contrast, distributions in the second quadrant (low-high) and fourth quadrant (high-low) were relatively rare.

### 4.5 Multi-scale driving mechanisms

In the field of ecological environment research, it is crucial to delve into the interaction mechanisms among various factors. To achieve a more comprehensive and precise analysis of their interdependencies, this study employs the geographical detector, a specialized analytical tool.

The q-statistic results from the single-factor Factor_detector (Table 6) indicate which factors have a more significant influence on the dependent variable.

**Table 6:** Q-values of impact factors across different years.

year	GDP	WET	LST	PRE	SLOPE	POP	NL
2000	0.000	0.792	0.461	0.135	0.734	0.005	0.033
2010	0.107	0.678	0.433	0.103	0.718	0.118	0.145
2020	0.034	0.700	0.400	0.027	0.690	0.146	0.141

In 2000, the q-values of the factors ranged from 0.00026 to 0.792. wet exhibited the highest q-value, followed by slope gradient, indicating that these two factors had strong explanatory power for the spatial heterogeneity of the study object. The q-value of lst was 0.461, representing a moderate level of explanation. The q-value of GDP was close to 0, suggesting its influence on the spatial distribution of the study object was negligible. Overall, wet and slope gradient were the core factors driving the formation of the spatial pattern of the study object in 2000, while the roles of GDP and population were minimal. The influence of the remaining factors decreased sequentially.

In 2010, the q-values ranged from 0.103 to 0.718. The q-value of slope gradient rose to the highest, with lst ranking second; both maintained strong explanatory power. The q-value of precipitation was relatively low, indicating weak explanatory power. During this phase, slope gradient and wet remained the core driving factors influencing the spatial pattern of the study object. The explanatory power of lst decreased slightly compared to 2000 but remained moderate. The influence of other factors on the spatial distribution was weak and relatively similar in degree.

In 2020, the differences in q-values among the factors became more pronounced. wet regained the highest q-value, while slope gradient remained second; their characteristic of strong explanatory power for spatial heterogeneity remained stable. Precipitation had the lowest q-value, indicating very weak explanatory power. In summary, wet and slope gradient were still the core driving factors of the spatial pattern in 2020. The explanatory power of lst continued to decline compared to 2010 but stayed at a moderate level. The explanatory power of population and nighttime light showed some fluctuation compared to 2010 and was comparable in degree, while the influence of GDP and precipitation was extremely weak.

**Fig 5:**
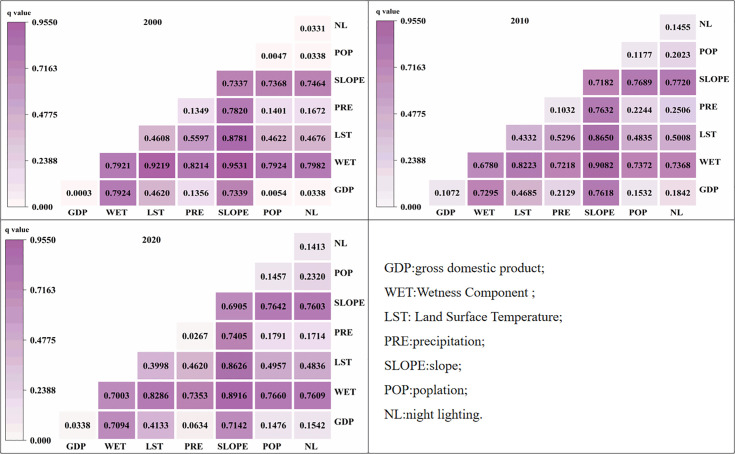
Results of geographical detector for various factors.

Interaction detection can reveal the interactions between different factors, as shown in Fig 5, The results showed significant interaction effects among factors.

The q-value of slope ∩ moisture was the highest in different years, indicating the most prominent interactive influence between slope and moisture. In 2000, 2010, and 2020, slope and moisture served as the main driving forces for NDVI, and their interactions with other factors also exhibited strong influences. Across these key time points (2000, 2010, 2020), slope and moisture emerged as dominant drivers affecting NDVI, thus influencing vegetation growth and recovery in the region.

Additionally, when interacting with other factors such as soil type, altitude, and intensity of human activities, slope and moisture also demonstrated strong influences. In single-factor detection, the impacts of GDP, population, and nighttime light were relatively low, indicating that their direct effects on NDVI were limited—likely due to the constraints of the special ecological environment in karst rocky desertification areas. Notably, however, their q-values increased when interacting with temperature and precipitation. This suggests that in the complex ecosystems of karst rocky desertification regions, factors with weak standalone effects can, through synergistic interactions with climatic factors, enhance their impact on the regional ecological environment and thus influence vegetation growth and recovery processes.

In conclusion, within the special ecological context of karst rocky desertification regions, slope and moisture, as key fundamental environmental factors, exert a more powerful influence when combined and interacting with other factors. This comprehensive influence alters regional water and heat conditions, soil physical and chemical properties, and material cycling and energy flow within ecosystems, ultimately exerting a significant impact on vegetation growth and recovery trends.

## 5. Discussion

This study realized a long-term dynamic coupling analysis of vegetation restoration and rocky desertification evolution in the karst region of Southwest China from 2000 to 2020. By integrating multi-source remote sensing data with a geographical detector model, it revealed the mutual feedback mechanisms of “ecological restoration - vegetation response - rocky desertification reversal”. The discussion unfolds across three dimensions: synergistic effects, driving mechanisms, and interactive effects.

### 5.1 Synergistic effects of rocky desertification spatiotemporal evolution and policy interventions

Rocky desertification in karst regions typically involves a complex interplay of geographical, climatic, social, and economic drivers. Consequently, ensuring long-term continuity and sustainability in governance represents a critical challenge in combating rocky desertification [35], while simultaneously serving as a fundamental catalyst for regional sustainable development.

Results show that From 2000 to 2020, the rocky desertification area in the karst region of Southwest China decreased from 154,200 km² to 71,500 km², indicating the significant effectiveness of ecological restoration projects. The core area of severe rocky desertification, located at the junction of Yunnan, Guizhou, and Guangxi provinces, consistently accounted for over 70% of the total degraded area (73.62% in 2020). However, this region exhibited a notably higher growth rate in NDVI (Normalized Difference Vegetation Index) compared to other areas. Notably, rocky desertification in Southwest China initially increased and then decreased during this period, with the total area declining from 154,200 km² in 2000–71,500 km² in 2020. The proportion of severe and extreme rocky desertification decreased from 10.52% to 6.14%, This trend is consistent with the findings of relevant studies [36].demonstrating the remarkable outcomes of ecological governance. These trends reflect a strong coupling between the vulnerability of karst ecosystems and human-driven restoration efforts under targeted policy interventions.

The study revealed that the rate of rocky desertification reduction accelerated after 2010, with an annual decline of 1.2%, closely linked to the long-term cumulative effects of the Grain for Green Program (launched in 2002) and the Rocky Desertification Comprehensive Control Project (initiated in 2007). Dynamic degree analysis further demonstrated that the expansion rate of non-desertified areas increased to 1.90% during 2010–2020, indicating a “threshold breakthrough effect” in ecological restoration—when vegetation coverage exceeds 0.6, the root system’s soil stabilization capacity and rainfall interception efficiency significantly improve [37], forming a positive feedback mechanism.

By 2020, extreme rocky desertification had nearly disappeared; however, scattered reversal zones persisted at the Guizhou-Yunnan border, highlighting the need to address localized ecological fragility. Spatial differentiation between stable and restored areas was quantified using a transition matrix, providing new evidence for differentiated governance strategies. Combined dynamic analysis further showed that non-desertified areas expanded at a rate of 1.41% during 2000–2020, while reversal zones accounted for 6.01% of the total area, underscoring the importance of vigilance against localized ecological regression risks.

### 5.2 Nonlinear relationship between vegetation cover structural optimization and rocky desertification reversal

For regions affected by rocky desertification, vegetation restoration serves as a key indicator for evaluating desertification reversal [38]. Furthermore, as karst ecosystems represent global biodiversity hotspots, restoring the stability and ecosystem service functions of these terrestrial systems promotes sustainable utilization of land resources.

Therefore, this study reveals the complex coupling mechanisms between vegetation restoration and rocky desertification reversal through NDVI dynamics.NDVI analysis revealed the complex coupling mechanism between vegetation restoration and rocky desertification reversal. From 2000 to 2020, the proportion of low-density vegetation pixels decreased from 27.6% to 9.3%, while high-density vegetation coverage surged from 12.8% to 34.5%, demonstrating a structural optimization pattern of “low-reduction and high-increase.” Regression analysis indicated a positive correlation between vegetation coverage and rocky desertification reduction, but this relationship exhibited nonlinear characteristics. When NDVI < 0.4, vegetation restoration had a weak inhibitory effect on rocky desertification (elasticity coefficient 0.35). However, the inhibitory effect significantly strengthened when NDVI ranged between 0.4 and 0.6, and it tended to saturate when NDVI > 0.6. This threshold effect aligns closely with the co-evolutionary dynamics of soil and vegetation in karst regions [39,40]. Low-coverage vegetation (NDVI < 0.4) primarily reduces soil loss through root-mediated surface soil consolidation, while medium-to-high coverage vegetation (NDVI > 0.4) enhances soil organic matter via litter layers, improving microhabitats [41], thereby establishing a positive feedback loop.

Meanwhile, Moran’s I index revealed a gradual decline in vegetation spatial aggregation, with a cumulative decrease of 0.034. However, the high-high aggregation clusters have expanded toward Yunnan and Guangxi, reflecting the spatial progression of ecological restoration from core zones to marginal areas. Nevertheless, attention must be given to the weakening aggregation intensity in regions like Chongqing, driven by urbanization pressures.

### 5.3 Interactive effects of multi-scale driving mechanisms

In studying ecological evolution in karst rocky desertification regions, the interaction between slope gradient and humidity emerged as a core driver of vegetation index dynamics. Based on long-term sequence analysis (2010–2020), their combined effect explained 46% of NDVI (Normalized Difference Vegetation Index) variation, highlighting the rigid constraints imposed by karst hydrological processes on vegetation recovery. Notably, while the nighttime light index exhibited low explanatory power in single-factor analyses, its interaction with precipitation and land surface temperature produced a sharp peak in q-value (0.48) by 2020. This phenomenon reveals the complex pathways through which urbanization indirectly impacts ecological restoration by altering local hydrological cycles. In karst desertification areas, population and GDP growth showed positive correlations with NDVI, confirming the pivotal role of intensive policy interventions in reshaping human-environment relationships in karst systems [42].Consequently, reconciling land-use conflicts with human activities presents a critical governance challenge in combating rocky desertification [43], while simultaneously constituting a key constraint on regional sustainable development.

Furthermore, vegetation coverage and rocky desertification reversal exhibit a “dual-threshold effect”: when coverage exceeds 0.6, the reversal rate accelerates significantly; however, once coverage surpasses 0.8, the carrying capacity of karst habitats approaches its limit, leading to diminishing marginal ecological benefits. This discovery provides a quantitative basis for precisely defining vegetation restoration targets. Spatially, NDVI changes displayed a gradient trend of increasing in central regions and decreasing in eastern and western zones, consistent with the restoration threshold effects of karst ecosystems. The primary drivers include:1)Central low-mountain areas (slope <25°, annual precipitation >1200 mm) exhibit stronger natural vegetation recovery capacity;2)Eastern and western high-altitude regions (>1500 m) and dry-hot valleys face dual constraints of long-term water stress and soil infertility [44], resulting in markedly delayed restoration.Spatial autocorrelation analysis revealed declining stability in NDVI high-value clusters, exemplified by the Wuling Mountain area of Chongqing. This trend directly correlates with forest habitat fragmentation driven by urban expansion.

## 6. Conclusions

This study is the first to explicitly propose the “dual threshold effect” between vegetation coverage (characterized by NDVI) and rocky desertification reversal in karst areas. Based on the Geodetector model, it systematically clarifies the “synergistic enhancement effect” of driving mechanisms for vegetation restoration in karst regions for the first time. It is also the first to quantify the spatial differentiation characteristics of rocky desertification “restoration areas, stable areas, and reversal areas” from 2000 to 2020. Additionally, it reveals the “threshold breakthrough effect” of ecological engineering initiatives for the first time, confirming that policies such as the Grain for Green Program and comprehensive rocky desertification control have accelerated restoration effects after breaking through ecological thresholds.The key findings are as follows:

[1] Remarkable Ecological Governance Outcomes: Policy-Driven Rocky Desertification Reversal.From 2000 to 2020, the rocky desertification area in Southwest China’s karst region decreased by 53.6%, with severe and extreme grades nearly eradicated. The continued implementation of the Grain for Green Program and Rocky Desertification Comprehensive Control Project drove the dynamic degree of non-desertified areas to increase from 0.55% (2000–2010) to 1.90% (2010–2020) through the mechanism of “vegetation reconstruction - habitat improvement - reverse succession”. Notably, the core area at the Yunnan-Guizhou-Guangxi border demonstrated the highest NDVI growth rate, further validating the effectiveness of targeted governance strategies.
[2] Vegetation Coverage Threshold Effects Reveal Optimization Pathways for Ecological Restoration.A nonlinear relationship exists between vegetation coverage and rocky desertification reversal. The probability of reversal increases significantly when coverage surpasses the 0.6 threshold, but marginal ecological gains diminish beyond the 0.8 threshold due to limitations in karst habitat carrying capacity. Geographical detector analysis identified slope-humidity interactions (q-value >0.4) as the dominant factor driving NDVI spatial differentiation. This underscores the necessity for governance strategies to prioritize synergistic terrain-hydrology regulation rather than relying solely on vegetation restoration alone.
[3] Spatial Heterogeneity Demands Zonal Governance and Systemic Regulation. Vegetation coverage exhibits strong spatial aggregation, yet the aggregation intensity shows a declining trend, indicating that engineering measures have weakened ecological heterogeneity. A “Three-Zone Regulation” strategy can be adopted:Restored zones (34.68%): Consolidate vegetation barriers;Reversal zones (6.01%): Implement soil improvement and industrial substitution;Stable zones (59.31%): Enhance NDVI dynamic monitoring.High-high aggregation clusters have expanded toward core governance areas like Yunnan and Guangxi, whereas regions such as Chongqing have experienced reduced forest patch connectivity due to built-up land expansion. The shrinkage of low-low aggregation zones correlates closely with ecological compensation policies, though localized reversal risks persist.

## Supporting information

S1 Data Minimum dataset. (ZIP)
